# The role of ultrasound and ultrasound-guided fine needle aspiration biopsy of lymph nodes in patients with skin tumours

**DOI:** 10.2478/raon-2013-0084

**Published:** 2014-01-22

**Authors:** Francesco Maria Solivetti, Fulvia Elia, Maria Giulia Santaguida, Antonino Guerrisi, Paolo Visca, Maria Cecilia Cercato, Aldo Di Carlo

**Affiliations:** 1 Radiologia e Diagnostica per Immagini, I.R.C.C.S Istituto Dermosifilopatico di Santa Maria e San Gallicano, Rome, Italy; 2 Sapienza Università di Roma, Dpt. Scienze Biotecnologie Medico-Chirurgiche, Rome, Italy; 3 Struttura di Anatomia ed Istologia Patologica e Citodiagnostica, I.R.C.C.S. Istituto Regina Elena, Rome, Italy; 4 Epidemiologia, Istituto Regina Elena, Rome, Italy; 5 Direzione Scientifica, I.R.C.C.S Istituto Dermosifilopatico di Santa Maria e San Gallicano, Rome, Italy

**Keywords:** skin tumours, ultrasound, fine needle aspiration biopsy, follow-up

## Abstract

**Background:**

The primary aim of this study was to evaluate the diagnostic accuracy of ultrasound (US) in the study of superficial lymph nodes during the follow-up of patients surgically treated for skin tumours. The secondary objective was to compare positive cytological results with histological reports.

**Patients and methods:**

From 2004 to 2011, 480 patients (male/female: 285/195; median age 57 years; prevalent skin tumour: melanoma) underwent US-guided fine-needle aspiration biopsy (FNAB) of suspicious recurrent lymph nodes. An expert radiologist first performed US testing of the lymph nodes, expressing either a negative or positive outcome of the test. Subsequently, US-guided FNAB was performed. FNAB positive patients were subjected to lymphadenectomy; the patients who tested negative underwent the follow-up.

**Results:**

The size of lymph nodes was ≤ 2 cm in 90% of cases. Out of the 336 (70%) US “positive” patients, 231 (68.8%) were FNAB positives. Out of the 144 (30%) US “negatives”, 132 (91.7%) were FNAB negatives. The sensitivity and specificity of the US were 95% and 55.7%, respectively; the negative predictive value was 91.7% and the positive predictive value was 68.8%. Definitive histological results confirmed FNAB positivity in 97.5% of lymphadenectomies.

**Conclusions:**

US is a sensitive method in the evaluation of superficial lymph nodes during the follow-up of patients with skin tumours. High positive predictive value of cytology was confirmed.

## Introduction

Ultrasound (US) still represents the main method for evaluating superficial lymph nodes in skin cancers, especially in cutaneous melanoma.^1^,^2^

Specifically, US is the preferred technique in determining superficial lymph node metastases, during the follow-up of patients with melanoma.^3^ In fact, US has proved to be superior to clinical examination in identifying lymph node metastases.^4^,^5^ Moreover, a recent meta-analysis has demonstrated that US examination is superior to other imaging techniques as computerized tomography (CT) and positron emission tomography (PET) in identifying secondary localizations in superficial lymph nodes.^6^

Although there is no general consensus on the utility and management of the follow-up of patients with cutaneous melanoma, the issue has been widely debated in literature.^3^,^7^–^9^ As there are no large-scale prospective studies^10^, some authors have even questioned the clinical efficacy of follow-up; as a matter of fact no evidence of prognostic advantage in terms of life expectancy nor improvement in the quality of life was reported.^8^,^9^ Nevertheless, many authors agree to plan frequent and long-term clinical checks, possibly associated with the use of US and other imaging techniques.^3^,^11^–^13^ While there are many studies on the use of US in lymph node pre-surgery stage of melanoma and in the identification of the sentinel lymph node^6^,^14^,^15^, there are relatively few studies on the diagnostic role of US as compared to fine-needle aspiration biopsy (FNAB) and the value of US-guided FNAB in the assessment of lymph node metastases from skin cancers during the follow-up.^16^,^17^

The aim of this study was to evaluate the diagnostic accuracy of US in the assessment of superficial lymph nodes, as compared to FNAB during the follow-up of patients previously surgically treated for skin tumours. The secondary aim was to evaluate the correlation between FNAB and the respective histology report in the subgroup of patients with a positive cytopathology result who subsequently underwent lymphadenectomy.

## Patients and methods

### Study population

All the patients in the follow-up at our institute (IRCCS San Gallicano Dermatological Institute, Rome, Italy), surgically treated for skin cancers and referred to the Radiology Service for US-guided FNAB of superficial lymph nodes, were considered eligible for the study. From January 2004 to January 2011, 480 patients underwent US-guided FNAB of superficial lymph nodes due to clinical evidence of enlarged lymph nodes or with US pattern suspected for metastasis. The population was characterized by a prevalence of males (1.46 male/female ratio) and by a median age of 57 years (range of 22–84 years). The prevalent skin tumour was melanoma (85%) of pathological stage I/II (Breslow thickness ≤ 1 mm, *N* = 327; 1.01–2.00 mm, *N* = 65; 2.01–4 mm, *N* = 16). A sentinel lymph node biopsy was performed in 19.8% of patients with melanoma (*N* = 81); the histological examination was negative in 56 patients and positive in the remaining 25.

The median time interval between the excision of the skin cancer and emergence of suspicious lymph node was 13 months (range of 12–16 months) (Table 1). Relevant medical history and clinical data of the patients were collected in a data sheet upon enrolment in the study (age, sex, ethnic group, weight, date of cutaneous neoplasm excision, and histology type). All instrumental examinations were performed by the same operator (FMS), a radiologist with 30 years of experience (about 2500 US examinations yearly performed in patients affected by dermatologic diseases). The operator performed a preliminary US evaluation of the lymph node concerned, with a yes/no assessment – negative or positive to the test – according to the detailed criteria in the following Ultrasound Image Analysis section; all data were reported on a specific data sheet. The FNAB examination was subsequently performed and the cytological specimens sent to the pathologist for cytological examination. In the case of inadequate material, the FNAB was repeated (7–10 days after the first examination). In the cases testing negative for neoplastic cells checks were performed to ensure that the cytological specimen contained a sufficient quantity of lymphocytes; sampling was repeated in the event of an insufficient quantity of cellular elements. All patients with a positive cytology report underwent surgical excision of the lymph node concerned and the related histology reports were acquired and used as standard for the comparison. All patients that tested negative to the cytological examination underwent clinical and instrumental monitoring in accordance with the Institute’s follow-up protocol.

### Ultrasound image analysis

US examinations were performed with a MyLab 70 XVC US system (Esaote s.p.a., Genoa, Italy) utilizing a LA 435 linear sensor, with frequency of between 6 and 18 MHz, or LA 523 (4–13 MHz). The examined lymph node was classified “negative” or “positive” on the basis of the radiologist’s opinion considering the US features.^18^ Specifically, lymph nodes that possessed at least one of the following characteristics were classified as “positive”: 1) round morphology (relation between the axial and longitudinal diameters < 2 in normal lymph nodes); 2) absence, attenuation or dislocation of the chillum; 3) eccentric cortical thickening or alteration of the contour of the lymph node; 4) lack of homogeneity in the cortical structure; 5) extracapsular extension; 6) one or more of the following vascular patterns: a) decrease in global vascularisation; b) cortical vascular structures of irregular calibre with a sharp interruption, tangential to the chillum rather than radial; c) absence of vascularisation in the chillum; d) the presence of peripheral vascular structures which penetrate into the cortical; e) highly or moderately resistant arterial signs or signs of a grossly altered morphology.^14^,^18^,^19^ In the absence of the above mentioned characteristics, the examination was classified as “negative”.

### FNAB, cytological and histological examination

The FNAB procedure was US-guided, with suitable settings for needle-tip identification. The freehand sampling technique was used, without any type of mechanical guidance. On average, the procedure took five-ten minutes subsequent to completion of the informed consent process, examination of previous imaging documentation and positioning of the patient on the bed. Position of the needle inside the target lesion was documented with imaging. Chiba-type needles were used, of a length between 17 and 60 mm, of variable calibre between 19 and 25 G, based on the operator‘s choice, with overall prevalence of 23 G. Sampling was nearly always capillary (98%), hardly ever applying a significant depression, either mechanical or with a syringe. Two samplings from two different areas of the same lymph node were usually carried out; three samplings were done in 50 cases and only one sampling in 22 cases, as the material obtained was sufficient for diagnostic purposes. The material obtained from the FNAB was treated according to two different methods: a) smeared on clean glass slide and fixed with an alcohol-based spray (until 2009); b) directly treated in liquid solution (PreservCyt®) (from 2009 onwards). The samples were sent to the Pathologic Anatomy Laboratory of I.F.O., where they were prepared according to the ThinPrep Pap Test (Thin-Prep®).^20^ All the cytology slides were stained using the Papanicolaou technique.

FNABs were considered adequate in presence of at least six groups of cells, each including 10–15 cells obtained from two aspirations of one lymph-node.

In the case of uncertain cytomorphology, immunocytochemical staining was carried out in order to reach a conclusive diagnosis. Specifically, paraffin-imbedded sections on glass slides were stained for HMB-45, MART-1, S100b protein markers in the case of suspected melanoma. In the case of suspected carcinoma metastasis, cytokeratin staining was performed.

The samples indicating massive necrosis, but in the absence of readable cells (2% of the cohort), were considered suspicious and were sent for surgical resection. The cases cytologically assessed as suspicious for cancer were considered positive for statistical purposes. The material obtained through surgical biopsy was fixed in formalin and included in paraffin; the 3-μm sections were stained with standard haematoxylin and eosin. In many cases, the histological diagnosis was backed up by immunohistochemical staining in order to clarify uncertain histomorphology.

### Statistical analysis

Continuous data were described with the mean or median value and the range; categorical data were presented with the frequency. The diagnostic accuracy of US was defined by calculating sensitivity, specificity, positive predictive value (PPV) and negative predictive value (PNV). Graph-Pad 5 software (GraphPad Co. La Jolla, CA-USA) was used to analyse statistical data.

## Results

**FIGURE 1.**
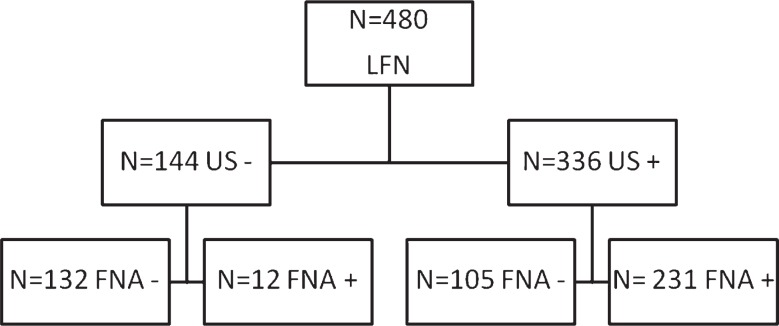
Flow-chart describing lymph-nodes analysed by ultrasound and FNAB cytology. LFN = lymph-nodes investigated; US− = ultrasound negative for lymph-node metastasis; US+ = ultrasound positive for lymph-node metastasis; FNAB = fine-needle aspiration biopsy; FNAB− = FNAB negative for lymph-node metastasis; FNAB+ = FNAB positive for lymph-node metastasis

Ninety percent of patients presented lymph nodes of ≤ 2 cm in size at US examination; in 129 of them the size was < 1.5 cm (Table 2). The prevalent lymph nodal stations were the axillary and inguinal (40 % in both cases). Of the 480 patients included in the study (Figure 1), 336 (70%) presented features suggesting recurrence (US+) at the US examination; in the remaining 144 patients (30%) the US pattern appeared non-suspicious, suggesting a reactive or inflammatory lymphadenopathy (US−). The absence, attenuation or dislocation of the chillum was the most frequent characteristic among positive lymph nodes; the round morphology of lymph nodes was the most important prognostic factor for the metastatic involvement.

The FNAB examination produced adequate cell material in nearly all cases - in 5 cases inadequate material at the first sampling made it necessary the repetition of the procedure, none at the second sampling. Neither complications nor significant sequel were reported. Upon cytological verification, 231 (68.8%) of the 336 US+ classified cases received confirmation and, therefore, represent the “true positives” (TP) for the test, whereas the remaining 105 (31.2%) with negative cytological reports, represent the “false positives” (FP). In the group of the 144 US− classified patients, 132 (91.7%) tested negative for neoplasm upon cytological examination and 12 (8.3%) tested positive, therefore representing the US “true negatives” (TN) and US “false negatives” (FN), respectively. According to these results, the sensitivity of US compared to cytological examination is 95%, with a specificity of 55.7%; the negative predictive value and the positive predictive value are equal to 91.7% and 68.8% respectively (Table 3).

FNAB positive patients (*N* = 243−231 US TP and 12 US FN) underwent surgical exeresis of the lymph node; definitive histological and cytological results were eventually compared. Of these, 237 (97.5%) received confirmation from the definitive histological examination, whereas in 6 cases (2.5%) the definitive histological examination resulted negative for neoplasm. Specifically, in the US− and FNAB+ cases, the histological examination high-lighted the presence of micrometastases or isolated tumour cells (ITC) of the lymph node, a condition which is generally harder to detect under US examination.

During the subsequent clinical and instrumental follow-up (average of 18 months, range of 12–40 months) no relapse in the same lymph node in none of the 132 TN patients (US− and FNAB−) was reported. In the group of 105 FP (US+ and FNAB−), 14 patients – representing 13.3% of this specific group and 4.2% of the suspicious US examination – had subsequent final evidence of lymph node metastases in the same location, four of whom after more than one year following FNAB.

## Discussion

Our study demonstrates that US, utilizing FNAB as a reference standard, is a sensitive method in the evaluation of lymph node metastatic involvement in skin tumours. Specifically, not only has US examination proved to be a valuable method for pre-operative melanoma staging^21^, but it is also a valid technique for assessing metastatic lymph nodes during the melanoma follow-up.

The high sensitivity observed in our cohort is also due to the wide neoplastic involvement of the lymph nodes as highlighted by the histological examination. All the US false negatives are in fact due to presence of micrometastasis and/or isolated tumour cells, findings that are more difficult to identify with US.

On the basis of the results, we can also infer that US is relatively inefficient in differentiating between inflammatory-induced and a neoplasm-induced structural alteration (55.7% specificity). Indeed, 105 out of 336 (31%) patients who were considered suspicious at the US examination, tested negative at the cytological examination. This finding suggests that US, either two-dimensional or combined with Doppler techniques for the study of lymph node vascularisation, does not seem to be conclusive in differentiating inflammatory-induced structural alteration from neoplasms. To this end, the need for technological improvements, new cultural acquisitions and the use of contrast-enhanced US is evident, with the possible use of other imaging techniques (i.e. computed tomography, magnetic resonance imaging).

A review of relevant literature shows that there have been many studies evaluating the ability of US in identifying lymph node metastases from skin tumours, especially melanoma. Considerable differences in the reported sensitivity and specificity values emerge from the results; these differences are ascribable to the wide variety of the study designs and the methods employed.^14^,^15^,^22^–^24^ Our results do not seem to be in accordance with the ones by other authors about the ability of US in evaluating lymph nodes. Specifically, Moehrle *et al*. report a US accuracy value close to 100% (100% sensitivity and 96% specificity), using few carefully-selected suspicious criteria on US. Moreover, a much smaller population was evaluated in said publications, generally analysing lymph nodes of larger sizes which is a decisive variable in highlighting the structural and architectural modification of the lymph nodes.^25^ In addition, other authors report a much lower sensitivity value (21 to 34%) 23,24; this represents a further example of the variability of the factors influencing the results and makes it difficult to compare the results from other studies.

As regards the follow-up of 105 patients suspicious upon US and with negative cytology reports, the presence of metastatic recurrence in the same lymph node location was detected after the first sampling in 14 cases (13.3%). The evidence of recurrence at FNAB in the same lymph node stations does not represent per se a valid parameter for the certification of the initial positivity of that lymph node; nevertheless, it clearly suggests that the number of correct diagnoses could be greater (72% of US+ cases).

It should be highlighted that performing a FNAB procedure there are significant probabilities of encountering areas not yet affected by the disease, especially lymph nodes of small sizes, as is the case with our cohort. Said element acquires a great significance, considering that in all our study cases the presence of lymphocytes in the material examined was documented, thus, confirming a correct execution of the cytological sample collection.

This evidence opens a discussion concerning the optimal methods for monitoring and the need for integrated diagnostic procedures in cases with suspicious US and negative cytology reports.

When comparing the results from US-guided FNAB with histology in pre-surgical assessment of the sentinel lymph node, some studies reported a positive predictive value greater than 65%.^15^,^26^ Another article reports a 100% FNAB sensitivity and specificity in a cohort of only 50 patients when compared to surgical biopsy.^16^ Other authors report FNAB values of sensitivity, specificity and positive predictive value of 90.9%, 67.2% and 82.6%, respectively. Besides, such cohort does not only refer to dermatological neoplasm and the inadequate samplings were discarded for statistical purposes.^27^ These results contrast slightly with those we obtained, but the methodological differences make it difficult to properly compare the data.

In our series, the number of inadequate samples (1%) is greatly inferior to the values reported in relevant literature (10% according to Basler *et al.*).^16^ This could be partly ascribable to the considerable specialization of the operators (sample taker and cytologist), as well as to the systematic sampling procedure of at least two different areas of the target lymph node; furthermore it is certainly also ascribable to the methods employed and the wide use of immunocytochemistry and special staining.

Moreover, the percentage of FNAB false positives as compared with histological results is very low, confirming the clinical significance of the positive result of fine needle aspiration.

Our study has methodological strong points, considering the high number of cases and the presence of a single operator which allows us to avoid the problem of inter-operator variability, which would otherwise occur; however, resorting to the cytological gold standard (imposed by the absence of histological verification of the lymph nodes with a negative histological report, for clearly ethical reasons) represents a limit of the study. It should be noted that the results have to be interpreted within a low-risk lymph node recurrence population like ours, mainly composed of melanomas in their early stages. Moreover, the level of diagnostic accuracy was obtained by a highly skilled operator. However, the specific characteristics of our population represent also a limit of the study since the cohort was selected on the basis of a suspicious metastatic involvement of superficial lymph nodes.

These elements have to be considered in the generalization of results.

## Conclusions

On the basis of the above, taking into consideration the great number of our records and in light of the difficulty in making a comparison with the cohort of other authors, we think that US, performed by expert operators, is of considerable value in excluding (except for micrometastases and ITC) the neoplastic involvement of superficial lymph nodes in the follow-up of patients with skin tumours. On the contrary, in about one third of the cases classified as suspicious upon US, the FNAB cytology demonstrates the absence of neoplastic cells; this highlights the existence of a fair number of false US positives, a diagnostic error determined by a difficult differential diagnosis between the inflammatory changes and the tumour.

A few of these patients showed later recurrence in the same lymph node location, thus suggesting the existence of a considerable percentage of false FNAB negatives, rightly related to a not yet massive neoplastic involvement of the lymph node. It is an empirically plausible element, given the small dimensions of the lymph nodes in our cohort. Hence the suggestion, limited to this group of patients, is for at least a closer clinical/US follow-up, even if this results in an inevitable increase in cost.

## Figures and Tables

**TABLE 1.** Patient characteristics (N = 480), tumour histotype and time interval between procedures

Age (years) – median (range)	57 (22–84)
Gender – no. (%)	
Male	285 (59.4)
Female	195 (40.6)
Weight (kg) – median (range)	72 (52–98)
Race or ethnic group – no. (%)	
White	453 (94.4)
Black	2 (0.4)
Other	25 (5.2)
Histotype– no. (%)	
Melanoma	408 (85)
Squamous Cell Carcinoma(SCC)	57 (11.8)
Other	15 (3.2)
Time interval between first surgical procedure and US (months)	
Median (range)	13 (10–15)

**TABLE 2.** Lymph-node characteristics (N = 480)

Lymph- node sites	
Axilla	192 (40%)
Inguinal Area	192 (40%)
Others (neck, popliteal and clavicular fossa)	96 (20%)
Lymph- node Size	
> 2 cm	48 (10 %)
1.5–2 cm	303 (63.1%)
< 1.5 cm	129 (26.9%)

**TABLE 3.** Sensitivity, specificity, positive predictive value (PPV) and negative predictive value (NPV) of ultrasound vs. fine-needle aspiration biopsy

**Imaging method**	**Specificity (%)**	**Sensitivity (%)**	**PPV (%)**	**NPV (%)**
US	55.7	95	68.75	91.7
